# Supplementary material for: Comparative Analysis of the GNAI Family Genes in Glioblastoma through Transcriptomics and Single-Cell Technologies
Source: Cancers (Basel). 2023 Oct 23;15(20):5112. doi: 10.3390/cancers15205112 (PMC10605456; doi:10.3390/cancers15205112)
Supplement: Supplementary file 1 [file cancers-15-05112-s001.zip › cancers-2599586-supplementary.pdf]

(A)

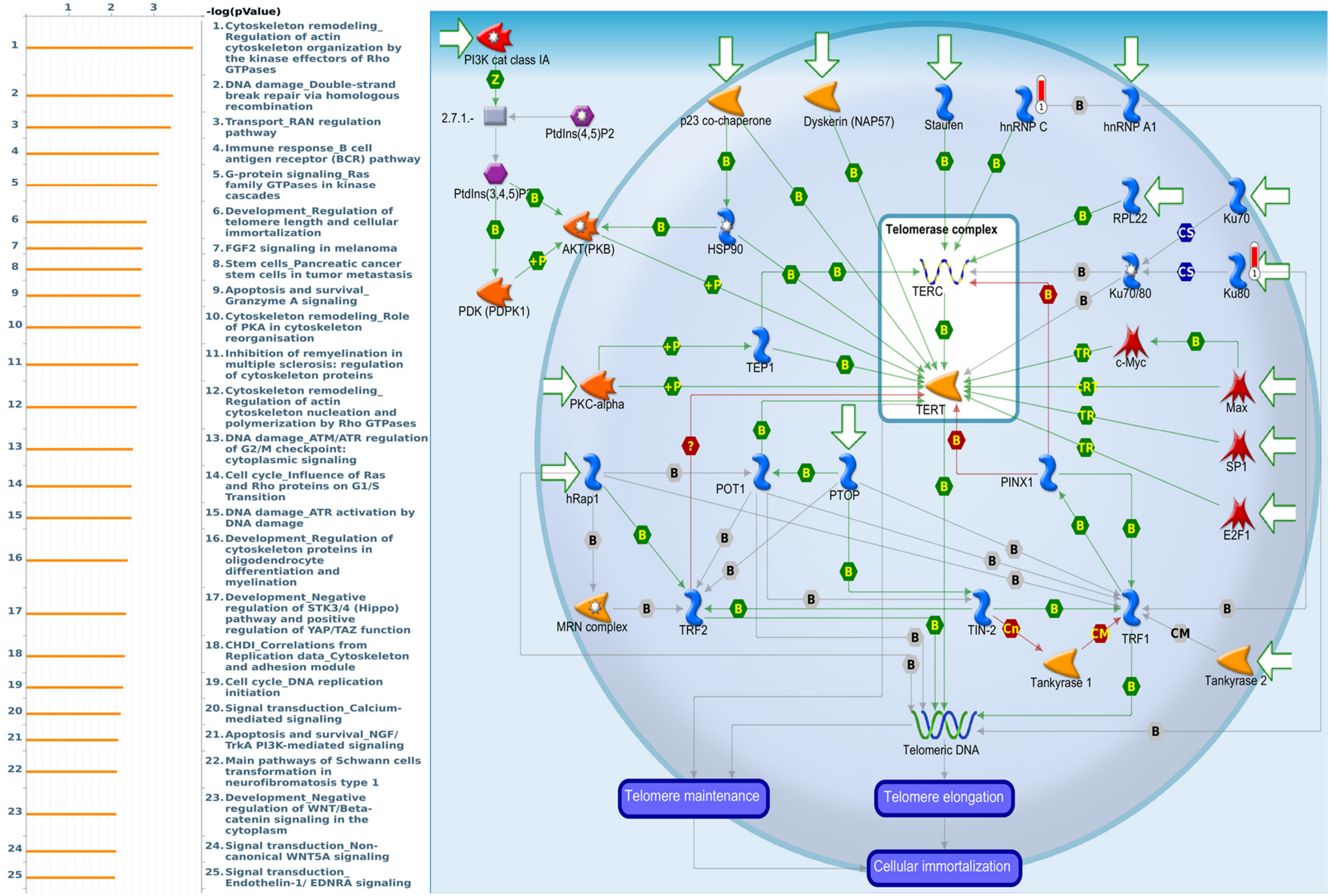
-log(pValue)
1
2
3
1. Cytoskeleton remodeling_ Regulation of actin cytoskeleton organization by the kinase effectors of Rho GTPases
2. DNA damage_Double-strand break repair via homologous recombination
3. Transport_RAN regulation pathway
4. Immune response_B cell antigen receptor (BCR) pathway
5. G-protein signaling_Ras family GTPases in kinase cascades
6. Development_Regulation of telomere length and cellular immortalization
7. FGF2 signaling in melanoma
8. Stem cells_Pancreatic cancer stem cells in tumor metastasis
9. Apoptosis and survival_ Granzyme A signaling
10. Cytoskeleton remodeling_Role of PKA in cytoskeleton reorganisation
11. Inhibition of remyelination in multiple sclerosis: regulation of cytoskeleton proteins
12. Cytoskeleton remodeling_ Regulation of actin cytoskeleton nucleation and polymerization by Rho GTPases
13. DNA damage_ATM/ATR regulation of G2/M checkpoint: cytoplasmic signaling
14. Cell cycle_Influence of Ras and Rho proteins on G1/S Transition
15. DNA damage_ATR activation by DNA damage
16. Development_Regulation of cytoskeleton proteins in oligodendrocyte differentiation and myelination
17. Development_Negative regulation of STK3/4 (Hippo) pathway and positive regulation of YAP/TAZ function
18. CHDI_Correlations from Replication data_Cytoskeleton and adhesion module
19. Cell cycle_DNA replication initiation
20. Signal transduction_Calcium-mediated signaling
21. Apoptosis and survival_NGF/ TrkA PI3K-mediated signaling
22. Main pathways of Schwann cells transformation in neurofibromatosis type 1
23. Development_Negative regulation of WNT/Beta-catenin signaling in the cytoplasm
24. Signal transduction_Non-canonical WNT5A signaling
25. Signal transduction_ Endothelin-1/ EDNRA signaling
PI3K cat class IA
2.7.1.-
PtdIns(4,5)P2
PtdIns(3,4,5)P3
AKT(PKB)
PDK (PDPK1)
p23 co-chaperone
Dyskerin (NAP57)
Staufen
hnRNP C
hnRNP A1
HSP90
RPL22
Ku70
Telomerase complex
TERC
Ku70/80
Ku80
TEP1
c-Myc
PKC-alpha
TERT
Max
SP1
hRap1
POT1
PTOP
PINX1
E2F1
MRN complex
TRF2
TIN-2
TRF1
CM
Tankyrase 1
Tankyrase 2
Telomeric DNA
Telomere maintenance
Telomere elongation
Cellular immortalization

(B)

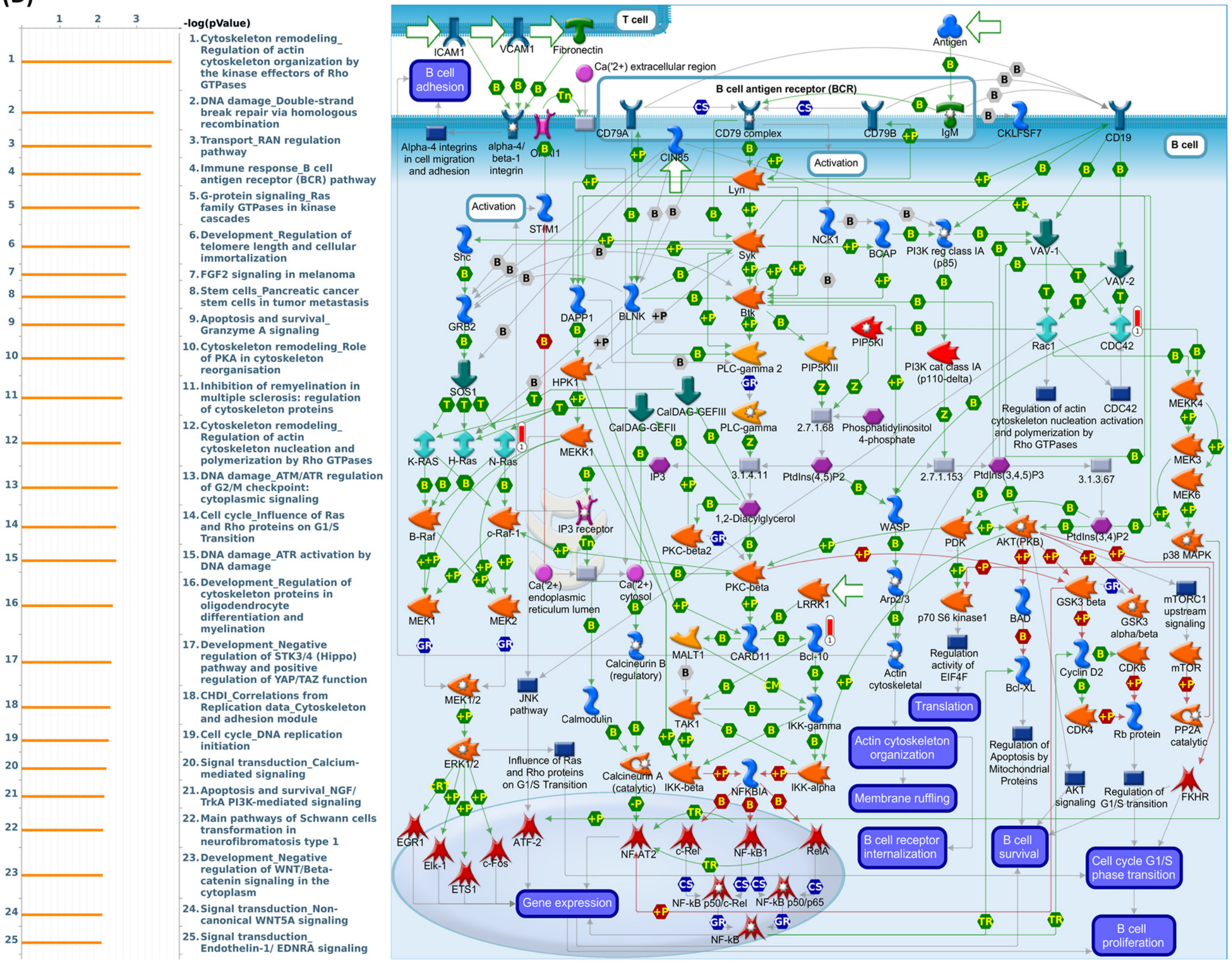

Supplementary Figure S1: **Expression of the *GNAI3* signaling pathway in GBM**. Using the MetaCore platform to analyze genes co-expressed with *GNAI3* from associated TCGA dataset, we found that **(A)** "Development Regulation of telomere length and cellular immortalization" and **(B)** "Immune response_B cell antigen receptor (BCR) pathway" were linked to GBM progression (with $p < 0.05$ set as the cutoff value).

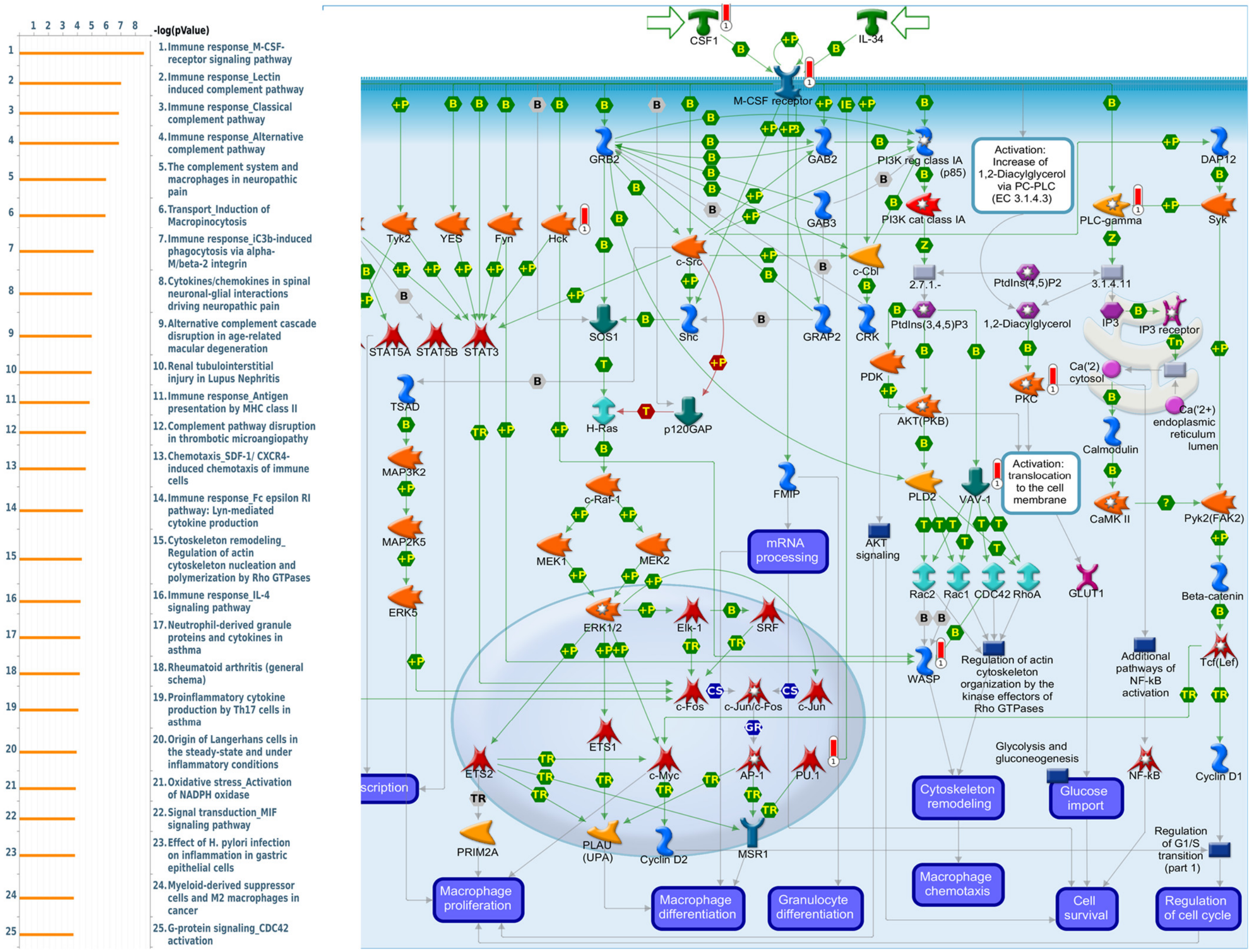

Supplementary Figure S2: **Expression of the *GNAI2* signaling pathway in GBM**. Using the MetaCore platform to analyze genes co-expressed with *GNAI2* from associated TCGA dataset, we found that "Immune response M-CSF-receptor signaling pathway" was linked to GBM (with $p < 0.05$ set as the cutoff value).

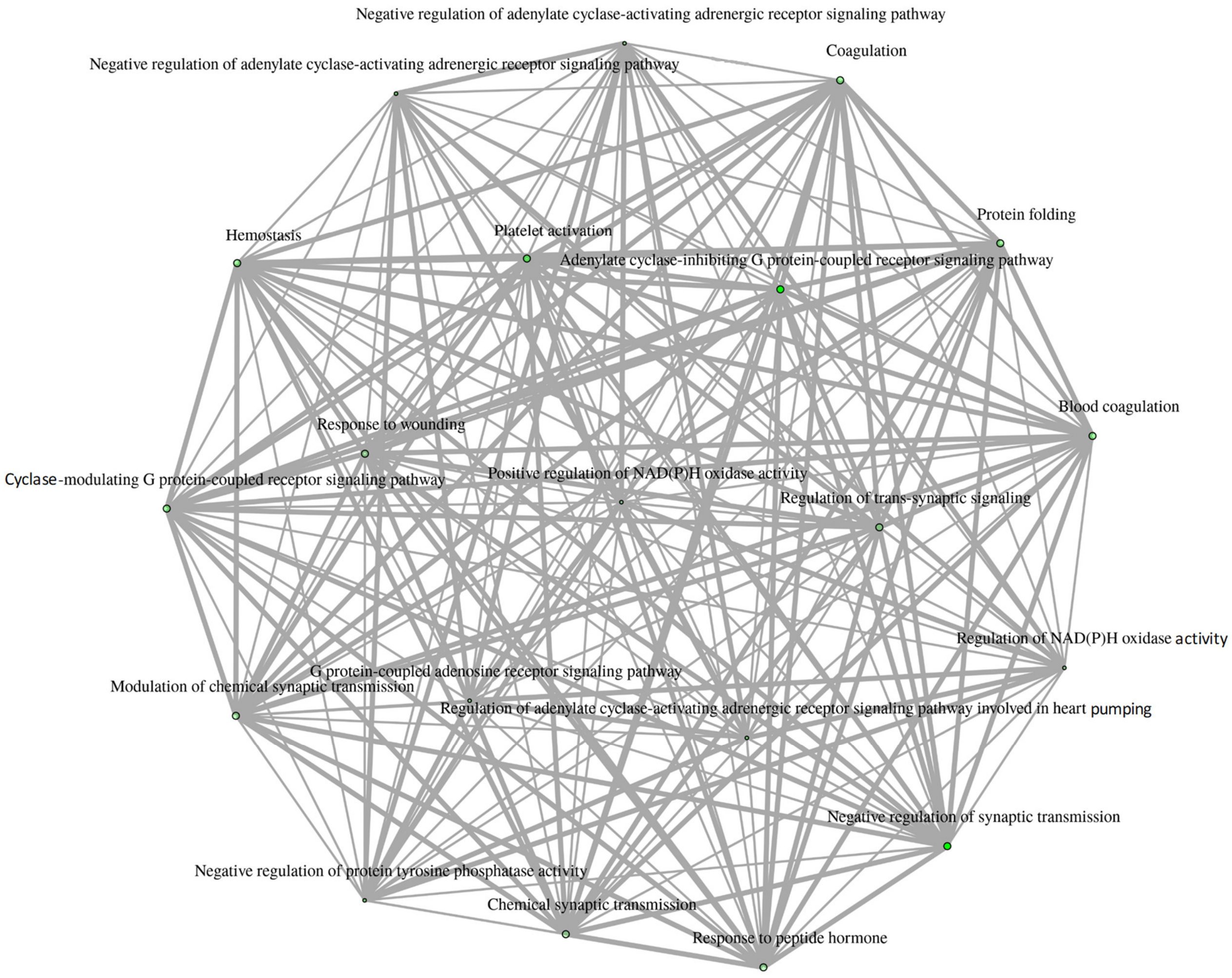

Supplementary Figure S3: **Pathway analysis**. Positively and negatively regulated pathways by *GNAI3*.

Supplementary Table S1. Top 163 genes used for MetaCore Analysis of *GNAI2*.

Sr. No.	Correlated Gene	Spearman's Correlation	p-Value
1.	SHISA5	0.597114628	2.24E-15
2.	MVP	0.594048181	3.38E-15
3.	KCNQ1	0.568125658	9.14E-14
4.	TWF2	0.56789482	9.40E-14
5.	SPI1	0.556022674	3.87E-13
6.	RAB43	0.555066131	4.32E-13
7.	TNFRSF1B	0.548874193	8.83E-13
8.	CD4	0.548275862	9.45E-13
9.	P2RY6	0.547228783	1.06E-12
10.	TYMP	0.544729177	1.41E-12
11.	CD74	0.544402456	1.46E-12
12.	VAV1	0.543378995	1.64E-12
13.	TAPBP	0.543272713	1.66E-12
14.	TGFB1	0.542619273	1.79E-12
15.	MYO1F	0.5425681	1.80E-12
16.	WAS	0.542225634	1.87E-12
17.	RASAL3	0.539269406	2.60E-12
18.	SIPA1	0.537742088	3.07E-12
19.	MAN1C1	0.537734215	3.07E-12
20.	SERPINA1	0.536171469	3.65E-12
21.	NAGA	0.533998583	4.62E-12
22.	FMNL1	0.533120768	5.08E-12
23.	CARD9	0.532971186	5.17E-12
24.	IL4R	0.532510628	5.43E-12
25.	BATF	0.532400409	5.50E-12
26.	IL2RB	0.532357109	5.52E-12
27.	CSF1R	0.531006928	6.39E-12
28.	ARHGAP45	0.528164856	8.66E-12
29.	GM2A	0.526610501	1.02E-11
30.	RHBDF2	0.525972288	1.09E-11
31.	A2M	0.522492521	1.58E-11
32.	AP1B1	0.521941427	1.67E-11
33.	GPR132	0.52132735	1.78E-11
34.	TRPV2	0.519634703	2.12E-11
35.	PLCG2	0.518878917	2.29E-11
36.	PALD1	0.518878917	2.29E-11
37.	ITGB2	0.518666352	2.35E-11
38.	VDR	0.518615689	2.36E-11
39.	RHOG	0.518571878	2.37E-11
40.	RIN3	0.515973862	3.09E-11
41.	GRN	0.515580224	3.22E-11

42.	PLEKHO2	0.515147221	3.37E-11
43.	CTSZ	0.514088333	3.75E-11
44.	DEF6	0.512190994	4.55E-11
45.	PRKCD	0.511738309	4.76E-11
46.	GIMAP1	0.511065187	5.09E-11
47.	CORO1A	0.510108644	5.61E-11
48.	HCK	0.50988427	5.74E-11
49.	ADPRH	0.509364667	6.04E-11
50.	CSF1	0.509041883	6.24E-11
51.	HCLS1	0.508514407	6.58E-11
52.	ARHGAP4	0.508297906	6.73E-11
53.	LILRB1	0.507778303	7.08E-11
54.	PPP1R18	0.50767202	7.16E-11
55.	SH3TC1	0.507593292	7.22E-11
56.	TMEM109	0.507375821	7.37E-11
57.	GPR84	0.506313966	8.19E-11
58.	DENND1C	0.50621162	8.28E-11
59.	PTPN6	0.503873406	1.04E-10
60.	ANO10	0.503231775	1.11E-10
61.	C3	0.502755472	1.16E-10
62.	TKT	0.502523225	1.19E-10
63.	CIITA	0.502082349	1.24E-10
64.	ABI3	0.501657219	1.30E-10
65.	TFE3	0.500440875	1.46E-10
66.	MYD88	0.500307038	1.48E-10
67.	PLXND1	0.498783656	1.71E-10
68.	STYXL2	0.498437254	1.77E-10
69.	DOCK2	0.497870414	1.87E-10
70.	TMEM102	0.497829572	1.88E-10
71.	FERMT3	0.49722091	1.99E-10
72.	C1R	0.496760353	2.08E-10
73.	ARPC1B	0.495599118	2.33E-10
74.	NCKAP1L	0.495158243	2.43E-10
75.	OSCAR	0.494229255	2.66E-10
76.	GNA15	0.494048668	2.70E-10
77.	STXBP2	0.492776728	3.05E-10
78.	LRRC25	0.492497245	3.13E-10
79.	SUSD6	0.49143442	3.46E-10
80.	NLRP3	0.491139191	3.56E-10
81.	LAIR1	0.489178869	4.28E-10
82.	TMC8	0.488588411	4.53E-10
83.	SLC15A3	0.488549047	4.55E-10
84.	TRPM2	0.488446701	4.59E-10

85.	LPAR5	0.48837191	4.62E-10
86.	RFTN1	0.488100299	4.74E-10
87.	FCGR3A	0.487694851	4.92E-10
88.	HLA-E	0.485628248	5.97E-10
89.	MYOF	0.484309558	6.74E-10
90.	ARHGAP30	0.484156038	6.84E-10
91.	CYB561D2	0.482341364	8.09E-10
92.	ADGRE1	0.482156353	8.22E-10
93.	TOR4A	0.482057944	8.30E-10
94.	IL21R	0.481715478	8.56E-10
95.	LGALS3BP	0.480963628	9.17E-10
96.	CCR5	0.480806172	9.31E-10
97.	CCR1	0.480782554	9.33E-10
98.	ALOX5	0.480160605	9.87E-10
99.	C1S	0.479794521	1.02E-09
100.	STAC3	0.479274917	1.07E-09
101.	PTPN7	0.47749567	1.26E-09
102.	NFAM1	0.47739726	1.27E-09
103.	WDFY4	0.477180759	1.29E-09
104.	VASP	0.477039049	1.31E-09
105.	PLCB2	0.476873721	1.33E-09
106.	SASH3	0.47668871	1.35E-09
107.	G6PD	0.474370178	1.67E-09
108.	RPS6KA1	0.474267832	1.68E-09
109.	PLA2G15	0.474039521	1.72E-09
110.	MSN	0.473626201	1.78E-09
111.	EBI3	0.473393954	1.82E-09
112.	DOK3	0.472716895	1.93E-09
113.	MAPKAPK3	0.472272083	2.01E-09
114.	ENG	0.471276177	2.19E-09
115.	CNPY3	0.470736892	2.30E-09
116.	TBXAS1	0.470732955	2.30E-09
117.	LGALS9	0.470567627	2.34E-09
118.	ITGAM	0.470421981	2.37E-09
119.	SCIN	0.470398362	2.37E-09
120.	THEMIS2	0.469563848	2.55E-09
121.	CAPZB	0.468686034	2.76E-09
122.	MAN2B1	0.46830814	2.85E-09
123.	CYTH4	0.468119194	2.90E-09
124.	ITGB5	0.467945993	2.94E-09
125.	LILRB4	0.467390962	3.09E-09
126.	CD14	0.466871359	3.23E-09
127.	HLA-DMA	0.46685955	3.24E-09

128.	CARD11	0.466517084	3.34E-09
129.	LAPTM5	0.465639728	3.60E-09
130.	MAPKAPK2	0.465320422	3.70E-09
131.	SYNGR2	0.464478035	3.98E-09
132.	LYL1	0.463907259	4.18E-09
133.	RAC2	0.462545268	4.71E-09
134.	IKZF1	0.462045347	4.91E-09
135.	ATG7	0.460742403	5.50E-09
136.	APOL3	0.460018107	5.85E-09
137.	CDCP1	0.459715006	6.00E-09
138.	STAT6	0.459215084	6.26E-09
139.	KCNK6	0.458845064	6.46E-09
140.	IL16	0.458845064	6.46E-09
141.	LPXN	0.458723036	6.53E-09
142.	ACAA1	0.458034168	6.92E-09
143.	CD44	0.457109117	7.49E-09
144.	LTC4S	0.456542277	7.86E-09
145.	CAPG	0.456506849	7.88E-09
146.	CD33	0.456373012	7.97E-09
147.	ARHGDIB	0.456148638	8.12E-09
148.	HCST	0.455967564	8.25E-09
149.	MAP4K1	0.455715635	8.43E-09
150.	FLVCR2	0.454995276	8.95E-09
151.	PCED1B	0.454885057	9.04E-09
152.	CXCL16	0.454396945	9.41E-09
153.	CYP2S1	0.453700205	9.98E-09
154.	CD68	0.453279011	1.03E-08
155.	DENND3	0.45160211	1.19E-08
156.	SIGLEC9	0.451562746	1.19E-08
157.	ICOSLG	0.451531255	1.20E-08
158.	DOK1	0.451511573	1.20E-08
159.	CMTM7	0.451090379	1.24E-08
160.	RASGRP4	0.451070698	1.24E-08
161.	SELPLG	0.450740041	1.28E-08
162.	ACTN1	0.450452685	1.31E-08
163.	TRADD	0.450003936	1.36E-08

Supplementary Table S2. Top 126 genes used for MetaCore Analysis of *GNAI3*.

Sr. No.	Correlated Gene	Spearman's Correlation	p-Value
1.	NRAS	0.771008503	8.12E-30
2.	DNTTIP2	0.627708235	2.93E-17
3.	LRRC42	0.61110455	3.27E-16
4.	DR1	0.588171154	7.33E-15
5.	KATNA1	0.586214769	9.45E-15
6.	RBM7	0.581657004	1.70E-14
7.	BCL10	0.57914108	2.33E-14
8.	NUP54	0.577786963	2.77E-14
9.	BZW1	0.57288616	5.10E-14
10.	PEX13	0.566186427	1.16E-13
11.	DDX20	0.561210833	2.10E-13
12.	GLO1	0.559348921	2.62E-13
13.	SNIP1	0.554121398	4.83E-13
14.	SERBP1	0.554117462	4.83E-13
15.	UTP11	0.553578177	5.14E-13
16.	HAT1	0.550740041	7.13E-13
17.	LRRC40	0.549122185	8.58E-13
18.	SIKE1	0.548181389	9.55E-13
19.	HNRNPLL	0.545000787	1.37E-12
20.	CDC42	0.542757046	1.76E-12
21.	NMD3	0.542649329	1.78E-12
22.	C1ORF109	0.5382302	2.91E-12
23.	CDC73	0.536675327	3.45E-12
24.	PPP1R8	0.53540781	3.97E-12
25.	SZRD1	0.532534247	5.42E-12
26.	SDE2	0.531762715	5.89E-12
27.	KPNA4	0.529589828	7.44E-12
28.	RPAP2	0.527586207	9.21E-12
29.	RBBP8	0.524303259	1.30E-11
30.	SF3A3	0.52128405	1.79E-11
31.	BRIX1	0.520382617	1.96E-11
32.	TXNDC12	0.520122815	2.02E-11
33.	PPIL4	0.519847268	2.08E-11
34.	FAF1	0.51515903	3.36E-11
35.	CAPZA1	0.514840183	3.47E-11
36.	HNRNPC	0.514773264	3.50E-11
37.	MCMBP	0.513836404	3.85E-11
38.	CMPK1	0.51209652	4.59E-11
39.	BCAS2	0.509474886	5.98E-11
40.	MARCHF7	0.508353015	6.69E-11
41.	RNF138	0.508199496	6.79E-11

42.	XRCC5	0.506077783	8.39E-11
43.	DHX40	0.505310187	9.05E-11
44.	BLOC1S5	0.502881436	1.15E-10
45.	RPF1	0.502558652	1.19E-10
46.	HSPA13	0.502334278	1.21E-10
47.	PPP1CB	0.501696583	1.29E-10
48.	GPX7	0.501428909	1.33E-10
49.	SNRNP40	0.500957035	1.39E-10
50.	ATG5	0.500826641	1.41E-10
51.	USP1	0.499917336	1.54E-10
52.	SEC22B	0.498771847	1.72E-10
53.	C3ORF38	0.497834987	1.88E-10
54.	PPIH	0.495638482	2.32E-10
55.	CNN3	0.495099197	2.44E-10
56.	APH1A	0.494327665	2.63E-10
57.	ABCB7	0.49355465	2.83E-10
58.	MAGOH	0.493347504	2.89E-10
59.	RIT1	0.493253031	2.92E-10
60.	NPM1	0.490674697	3.72E-10
61.	PAIP1	0.489497717	4.16E-10
62.	METAP2	0.48611636	5.70E-10
63.	SMNDC1	0.486002204	5.76E-10
64.	DESI2	0.485923477	5.81E-10
65.	SLC25A24	0.485581011	5.99E-10
66.	SLC16A1	0.485045662	6.30E-10
67.	YTHDF2	0.484130928	6.86E-10
68.	TBCE	0.483148323	7.51E-10
69.	CDK2	0.482782239	7.76E-10
70.	TMED5	0.482605102	7.89E-10
71.	TBC1D23	0.481746969	8.54E-10
72.	CENPL	0.481595892	8.66E-10
73.	WDR43	0.481215557	8.96E-10
74.	AKIRIN1	0.480266887	9.78E-10
75.	AK2	0.480164541	9.87E-10
76.	MRPL37	0.479786648	1.02E-09
77.	E2F6	0.479723666	1.03E-09
78.	TCEAL9	0.477582271	1.25E-09
79.	GNG12	0.476708392	1.35E-09
80.	COPB1	0.475680995	1.48E-09
81.	GLMN	0.475590926	1.49E-09
82.	KTI12	0.475299165	1.53E-09
83.	TMCO1	0.474145804	1.70E-09
84.	CSDE1	0.474039521	1.72E-09

85.	TMEM38B	0.473614391	1.78E-09
86.	GPN1	0.471256495	2.20E-09
87.	THG1L	0.471236813	2.20E-09
88.	TIPRL	0.469752795	2.51E-09
89.	GORAB	0.469697685	2.52E-09
90.	ITGB3BP	0.469469375	2.57E-09
91.	ACTR3	0.469323729	2.61E-09
92.	WDR3	0.468146749	2.89E-09
93.	DEK	0.467678318	3.01E-09
94.	COA7	0.46742639	3.08E-09
95.	RLF	0.466131318	3.45E-09
96.	ZNF644	0.464706345	3.90E-09
97.	RBBP4	0.464541017	3.96E-09
98.	POLR3C	0.464399307	4.01E-09
99.	NAA50	0.464226106	4.07E-09
100.	CPSF3	0.463808849	4.22E-09
101.	GNL2	0.463804913	4.22E-09
102.	ZZZ3	0.463793103	4.23E-09
103.	ADH5	0.462368131	4.78E-09
104.	RBM34	0.461986301	4.94E-09
105.	SH3GLB1	0.461946937	4.96E-09
106.	CENPQ	0.461824909	5.01E-09
107.	BTF3L4	0.461006141	5.37E-09
108.	RSL1D1	0.460651866	5.54E-09
109.	MRPS30	0.459963451	5.87E-09
110.	ASNSD1	0.459061565	6.34E-09
111.	EIF3M	0.458490789	6.66E-09
112.	YRDC	0.458207369	6.82E-09
113.	CALU	0.457778303	7.08E-09
114.	RRAGC	0.456317903	8.01E-09
115.	DDX59	0.455798748	8.37E-09
116.	ACADM	0.455573925	8.53E-09
117.	MFSD14A	0.455239332	8.77E-09
118.	PRPF4	0.454420564	9.39E-09
119.	TRMT10C	0.454117462	9.64E-09
120.	MIER1	0.454003307	9.73E-09
121.	PLRG1	0.453975752	9.75E-09
122.	TCEANC2	0.453798614	9.90E-09
123.	ZPR1	0.452633444	1.09E-08
124.	DUSP11	0.450358211	1.32E-08
125.	PRIM2	0.450314911	1.32E-08
126.	MYNN	0.450208629	1.33E-08

Supplementary Table S3. Top Correlated Pathways and C0-expressive genes linked with *GNAI2*.

Sr. No.	Maps	p-value	Network Objects from Active Data
1	Immune response_M-CSF-receptor signaling pathway	2.608E-09	PLC-gamma, PU.1, WASP, CSF1, VAV-1, M-CSF receptor, Hck, PKC
2	Immune response_Lectin induced complement pathway	1.028E-07	C3, C3b, C3c, C3dg, iC3b, C3a
3	Immune response_Classical complement pathway	1.469E-07	C3, C3b, C3c, C3dg, iC3b, C3a
4	Immune response_Alternative complement pathway	1.469E-07	C3, C3b, C3c, C3dg, iC3b, C3a
5	The complement system and macrophages in neuropathic pain	1.182E-06	C3, PKC-delta, TNF-R2, C3b, TGF-beta 1, MyD88, C3a
6	Transport_Induction of Macropinocytosis	1.289E-06	PLC-gamma, CORO1A(CLABP, TACO), RhoG, CSF1, M-CSF receptor, PKC
7	Immune response_iC3b-induced phagocytosis via alpha-M/beta-2 integrin	8.711E-06	ITGB2, VAV-1, PLC-gamma 2, iC3b, PKC
8	Cytokines/chemokines in spinal neuronal-glial interactions driving neuropathic pain	1.118E-05	PLC-gamma, P2Y6, TNF-R2, CSF1, M-CSF receptor, PKC
9	Alternative complement cascade disruption in age-related macular degeneration	1.185E-05	C3, C3b, iC3b, C3a
10	Renal tubulointerstitial injury in Lupus Nephritis	1.192E-05	TNF-R2, CSF1, M-CSF receptor, TGF-beta 1, CD4
11	Immune response_Antigen presentation by MHC class II	1.672E-05	CD74, PKC-delta, CD4, PKC, MyD88, HCLS1
12	Complement pathway disruption in thrombotic microangiopathy	3.013E-05	C3, C3b, PKC, C3a
13	Chemotaxis_SDF-1/ CXCR4-induced chemotaxis of immune cells	3.097E-05	ITGB2, WASP, SFK, VAV-1, PLC-gamma 2
14	Immune response_Fc epsilon RI pathway: Lyn-mediated cytokine production	4.936E-05	CARD9, PLC-gamma, PKC-delta, VAV-1, PKC
15	Cytoskeleton remodeling_Regulation of actin cytoskeleton nucleation and polymerization by Rho GTPases	5.835E-05	WASP, FMNL1, Rac1-related, DRF
16	Immune response_IL-4 signaling pathway	7.153E-05	PLC-gamma, IL4RA, PKC-delta, PLC-gamma 2, PKC
17	Neutrophil-derived granule proteins and cytokines in asthma	7.498E-05	Hck, Alpha-defensin, TGF-beta 1, PKC
18	Rheumatoid arthritis (general schema)	8.122E-05	TGF-beta, TNF-R2, CSF1, CD4
19	Proinflammatory cytokine production by Th17 cells in asthma	1.022E-04	C3, TGF-beta 1, CD4, C3a
20	Origin of Langerhans cells in the steady-state and under inflammatory conditions	1.361E-04	CSF1, M-CSF receptor, TGF-beta 1
21	Oxidative stress_Activation of NADPH oxidase	1.556E-04	PLC-gamma, PKC-delta, VAV-1, PKC
22	Signal transduction_MIF signaling pathway	1.772E-04	CD74, PU.1, CD74-ICD, SFK

23	Effect of H. pylori infection on inflammation in gastric epithelial cells	1.772E-04	PLC-gamma, CD74, PLC-gamma 2, MyD88
24	Myeloid-derived suppressor cells and M2 macrophages in cancer	2.135E-04	IL4RA, CSF1, M-CSF receptor, TGF-beta 1
25	G-protein signaling_CDC42 activation	2.267E-04	CSF1, VAV-1, M-CSF receptor, PKC
26	B-regulatory cells and tumor cells intercellular interaction	2.549E-04	IL4RA, TNF-R2, VDR, TGF-beta 1
27	Immune response_CD16 signaling in NK cells	2.856E-04	WASP, VAV-1, PLC-gamma 2, SHP-1
28	Inhibition of neutrophil migration by proresolving lipid mediators in COPD	3.187E-04	ITGB2, TNF-R2, VAV-1, PKC
29	Development_Transcription factors in segregation of hepatocytic lineage	3.494E-04	A2M, TGF-beta 1, Alpha 1-antitrypsin
30	Development_G-CSF-induced myeloid differentiation	3.494E-04	PU.1, ITGB2, SHP-1
31	Immune response_IL-6 signaling pathway via MEK/ERK and PI3K/AKT cascades	3.736E-04	PKC-delta, Proepithelin, VAV-1, Hck
32	G-protein signaling_Rac1 activation	3.736E-04	Rho GTPase, CORO1A(CLABP, TACO), Rac1-related, VAV-1
33	COVID-19 associated coagulopathy	4.137E-04	C3, A2M, C3b, PKC
34	Cell adhesion_Integrin inside-out signaling in neutrophils	4.795E-04	ITGB2, PKC-delta, Hck, PLC-gamma 2
35	Production and activation of TGF-beta in airway smooth muscle cells	5.541E-04	TGF-beta, TGF-beta 1, MyD88
36	Immune response_Th17, Th22 and Th9 cell differentiation	7.635E-04	PU.1, TGF-beta 1, CD4
37	Macrophage-induced immunosuppression in the tumor microenvironment	1.039E-03	ILT2, CSF1, M-CSF receptor, TGF-beta 1
38	Immune response_IL-13 signaling via JAK-STAT	1.089E-03	IL4RA, PKC-delta, SHP-1
39	Inhibition of remyelination in multiple sclerosis: regulation of cytoskeleton proteins	1.089E-03	TGF-beta, WASP, VAV-1
40	SLE genetic marker-specific pathways in B cells	1.122E-03	PLC-gamma, PKC-delta, VAV-1, SHP-1
41	COVID-19: immune dysregulation	1.164E-03	CSF1, TGF-beta 1, CD4, MyD88
42	Macrophage and dendritic cell phenotype shift in cancer	1.164E-03	CSF1, M-CSF receptor, TGF-beta 1, SHP-1
43	Immune response_Naive CD4+ T cell differentiation	1.239E-03	PU.1, TGF-beta 1, CD4
44	Th9 cells in asthma	1.320E-03	PU.1, TGF-beta, TGF-beta 1
45	Signal transduction_Delta-type opioid receptor signaling in non-neuronal cells	1.320E-03	PKC-delta, VAV-1, PKC
46	Signal transduction_Adenosine A3 receptor signaling pathway	1.403E-03	Rho GTPase, PKC-delta, PKC
47	Th17 cells in CF (mouse model)	1.490E-03	TGF-beta 1, CD4, MyD88
48	Immune response_B cell antigen receptor (BCR) pathway	1.656E-03	PLC-gamma, WASP, VAV-1, PLC-gamma 2
49	Immune response_T cell subsets: cell surface markers	1.769E-03	IL4RA, TGF-beta 1, CD4
50	Immune response_Inhibitory PD-1 signaling in T cells	1.869E-03	VAV-1, CD4, SHP-1

Supplementary Table S4. Top Correlated Pathways and C0-expressive genes linked with *GNAI3*.

Sr. No.	Maps	p-value	Network Objects from Active Data
1	Cytoskeleton remodeling_Regulation of actin cytoskeleton organization by the kinase effectors of Rho GTPases	1.230E-04	Cdc42 subfamily, CDC42, MLCP (cat)
2	DNA damage_Double-strand break repair via homologous recombination	3.690E-04	RBBP8 (CtIP), NARF, Ku80
3	Transport_RAN regulation pathway	4.132E-04	NUP54, Importin (karyopherin)-alpha
4	Immune response_B cell antigen receptor (BCR) pathway	8.123E-04	CDC42, Bcl-10, N-Ras
5	G-protein signaling_Ras family GTPases in kinase cascades	8.705E-04	CDC42, N-Ras
6	Development_Regulation of telomere length and cellular immortalization	1.579E-03	hnRNP C, Ku80
7	FGF2 signaling in melanoma	1.958E-03	CDC42, N-Ras
8	Stem cells_Pancreatic cancer stem cells in tumor metastasis	2.059E-03	CDC42, MLCP (cat)
9	Apoptosis and survival_Granzyme A signaling	2.163E-03	hnRNP C, Ku80
10	Cytoskeleton remodeling_Role of PKA in cytoskeleton reorganisation	2.163E-03	CDC42, MLCP (cat)
11	Inhibition of remyelination in multiple sclerosis: regulation of cytoskeleton proteins	2.487E-03	CDC42, MLCP (cat)
12	Cytoskeleton remodeling_Regulation of actin cytoskeleton nucleation and polymerization by Rho GTPases	2.716E-03	Cdc42 subfamily, CDC42
13	DNA damage_ATM/ATR regulation of G2/M checkpoint: cytoplasmic signaling	3.329E-03	MLCP (cat), PP1-cat
14	Cell cycle_Influence of Ras and Rho proteins on G1/S Transition	3.590E-03	CDC42, MLCP (cat)
15	DNA damage_ATR activation by DNA damage	3.590E-03	RBBP8 (CtIP), SNIP1
16	Development_Regulation of cytoskeleton proteins in oligodendrocyte differentiation and myelination	4.430E-03	CDC42, MLCP (cat)
17	Development_Negative regulation of STK3/4 (Hippo) pathway and positive regulation of YAP/TAZ function	4.881E-03	NEDD4, MLCP (cat)
18	CHDI_Correlations from Replication data_Cytoskeleton and adhesion module	5.193E-03	CDC42, MLCP (cat)
19	Cell cycle_DNA replication initiation	5.678E-03	MCMBP, Importin (karyopherin)-alpha
20	Signal transduction_Calcium-mediated signaling	6.531E-03	Bcl-10, MLCP (cat)
21	Apoptosis and survival_NGF/ TrkA PI3K-mediated signaling	7.438E-03	CDC42, MLCP (cat)
22	Main pathways of Schwann cells transformation in neurofibromatosis type 1	8.008E-03	CDC42, N-Ras
23	Development_Negative regulation of WNT/Beta-catenin signaling in the cytoplasm	8.203E-03	FAF1, PP1-cat
24	Signal transduction_Non-canonical WNT5A signaling	8.399E-03	CDC42, MLCP (cat)
25	Signal transduction_Endothelin-1/ EDNRA signaling	8.798E-03	CDC42, MLCP (cat)

26	Immune response_CD28 signaling	9.413E-03	CDC42, Bcl-10
27	Immune response_Fc epsilon RI pathway: Lyn-mediated cytokine production	9.413E-03	CDC42, Bcl-10
28	Development_The role of GDNF ligand family/ RET receptor in cell survival, growth and proliferation	1.048E-02	CDC42, N-Ras
29	Chemotaxis_Lysophosphatidic acid signaling via GPCRs	1.990E-02	Rho GTPase, CDC42
30	Cell cycle_Nucleocytoplasmic transport of CDK/Cyclins	2.349E-02	Importin (karyopherin)-alpha
31	Cytoskeleton remodeling_Substance P mediated membrane blebbing	2.681E-02	MLCP (cat)
32	Transition of Smoldering multiple myeloma to active multiple myeloma (schema)	2.846E-02	N-Ras
33	IL-1 production in melanoma	2.846E-02	N-Ras
34	Multiple myeloma (general schema)	3.011E-02	N-Ras
35	Anti-apoptotic pathways in endoplasmic reticulum stress response in multiple myeloma	3.176E-02	PP1-cat
36	Immune response_ETV3 affect on CSF1-promoted macrophage differentiation	3.176E-02	DDX20
37	Inhibition of GSK3 beta by lithium in major depressive disorder	3.668E-02	PP1-cat
38	Cytoskeleton remodeling_CDC42 in cellular processes	3.832E-02	CDC42
39	G-protein signaling_Cross-talk between Ras-family GTPases	3.832E-02	CDC42
40	G-protein signaling_CDC42 inhibition	3.995E-02	CDC42
41	Development_ROBO2, ROBO3 and ROBO4 signaling pathways	4.158E-02	CDC42
42	Defective macrophage-mediated bacterial phagocytosis in COPD	4.158E-02	CDC42
43	Cell adhesion_Classical cadherin-mediated cell adhesion	4.321E-02	CDC42
44	Brca1 in ovarian cancer	4.484E-02	RBBP8 (CtIP)
45	Neurophysiological process_GABA-A receptor life cycle	4.484E-02	PP1-cat
46	Role of Th17 cells in asthma	4.646E-02	MLCP (cat)
47	Rho-dependent regulation of normal and asthmatic smooth muscle contraction	4.646E-02	MLCP (cat)
48	Regulation of Beta-catenin activity in melanoma	4.646E-02	N-Ras
49	DNA damage_Brca1 as a transcription regulator	4.970E-02	RBBP8 (CtIP)
50	Inhibition of Ephrin receptors in colorectal cancer	4.970E-02	CDC42
